# Enhancing Medical Education through the “Distribute, Discuss, and Develop” Method: A Comparative Study of Small-Group Discussions

**DOI:** 10.7759/cureus.59012

**Published:** 2024-04-25

**Authors:** Arunita T Jagzape, Mahendra Kumar, Nilabh Ghritlahre

**Affiliations:** 1 Physiology, All India Institute of Medical Sciences, Raipur, Raipur, IND; 2 Physiology, Narayan Medical College and Hospital, Sasaram, IND; 3 Physiology, Pt. Jawahar Lal Nehru Memorial Medical College, Raipur, IND

**Keywords:** small-group discussions, class-average normalized gain, communication skills, effect size, feedback

## Abstract

Background

Small-group discussions (SGDs) are pivotal in medical education, facilitating the development of critical thinking, communication skills, and teamwork. However, traditional SGDs face challenges such as scalability and maintaining student engagement. This study aims to evaluate the “Distribute, Discuss, and Develop” (3D) method for enhancing learning outcomes in medical education.

Methods

A single-blinded interventional study was conducted with 125 first-year Bachelor of Medicine and Bachelor of Surgery students, who were divided into intervention and control groups through random assignment. The intervention group employed the 3D method across two thematic units: hematology and muscle nerve physiology. The study assessed learning outcomes using pre- and posttests, class-average normalized gain (“g”), and feedback questionnaires to capture student perceptions of interaction, communication enhancement, and session summarization.

Results

The intervention group showed significantly improved learning outcomes in both thematic units, with larger effect sizes (hematology: 1.55; muscle nerve physiology: 1.4) compared to the control group. The normalized gain “g” indicated a medium effectiveness level for the intervention group in both themes, suggesting enhanced learning. Feedback questionnaires revealed higher satisfaction levels within the intervention group regarding interaction, communication skills, and session summarization.

Conclusions

The 3D method addresses the challenges faced by traditional SGDs, providing a scalable and engaging approach to medical education. By fostering more effective student-centered learning, the method enhances the comprehension of complex physiological concepts and improves communication skills. The 3D method significantly improves learning outcomes, interaction, and communication skills in medical education. This innovative approach to SGDs offers a promising strategy for enhancing the educational experience in medical schools, supporting the development of more articulate and professionally competent medical graduates.

## Introduction

Interacting in small groups enables students to refine their understanding through discussion, apply their knowledge to new situations, and reflect on their feelings and attitudes [1]. Medical schools worldwide employ various instructional techniques, including the traditional lecture method and small-group discussion (SGD) methods, such as problem-based learning, seminars, symposiums [2], tutorials [3], and discussions [4].

Groups of four to six members are generally considered ideal for learning purposes. SGDs foster skills in listening, presenting ideas, and teamwork and enhance critical thinking and student engagement. SGDs allow students to monitor their learning, promoting independence and self-direction. However, increasing the group size may reduce interaction opportunities, though dividing large groups into smaller ones can preserve the benefits of SGDs [4]. The discussion’s conduct can positively or negatively influence students’ interests [5]. Given the need to accommodate growing student numbers while maintaining the effectiveness of group discussions, this study introduces an approach called the “Distribute, Discuss, and Develop” (3D) method in teaching physiology. The study objectives are to compare the performance of students participating in SGDs using the 3D method with those in traditional SGDs and gather perceptions of students participating in SGDs using the 3D method with those in traditional SGDs, thereby assessing if modifications in SGDs enhance learning. The research question is: "Does modifications in SGD improve learning as compared to the traditional SGDs?"

## Materials and methods

This interventional study included 125 first-year Bachelor of Medicine and Bachelor of Surgery (MBBS) students from the 2020-21 batch. We divided these students into three batches of 42, 42, and 41, respectively, using convenience sampling. Each batch was then split into two groups of 20 to 21 students. Through random assignment, we designated one group from each batch to the intervention group (Group A) and the other to the control group (Group B). The study began with a focus on hematology and subsequently crossed over to muscle nerve physiology. Employing a single-masked approach, the person analyzing the study’s results was unaware of the participants’ group assignments. All study participants received information about the study procedures and gave written informed consent, which assured confidentiality. The All India Institute Of Medical Sciences, Raipur (AIIMS Raipur) Institutional Ethics Committee approved the study design (Approval No. 854/IEC-AIIMSRPR/2019).

In the hematology section, the control and intervention groups included 46 students each, while in the muscle nerve physiology section, each group comprised 54 students. The SGDs allocated two hours per session, with topics announced in advance to allow students to prepare. In implementing the 3D strategy, tutorials began by dividing 20 to 21 students into five smaller subgroups of four to five students each. These subgroups were arranged to face each other, enhancing communication based on the principle that circular seating facilitates interaction (4). The initial session introduced group dynamics, instructions, and the mind map technique (15 min). We then divided the central topic into five subtopics reflecting the learning objectives, displaying these on a greenboard. For hematology, these subtopics included erythropoiesis, plasma proteins, jaundice, white blood cells and immunity, and hemostasis. Muscle nerve physiology subtopics covered the classification of nerve and functional anatomy of the skeletal muscle, neuromuscular junction, and myasthenia gravis; drugs affecting neuromuscular junction, excitation-contraction coupling, and muscle contraction; and energetics and oxygen debt.

The “Discuss” phase had all groups thoroughly discuss the prepared subtopics, with educational materials accessible for deeper exploration (30 min). During the “Develop” phase, a randomly chosen member from any group would explain concepts on the board using mind maps or concept maps, fostering active participation and discussion among all students. This included clarifying questions from peers and the tutor, with facilitators encouraging participation from quieter members (45 min). The facilitator summarized the topics and points discussed, concluding with a posttest and feedback collection ( 20 min). Attendance was recorded to ensure efficient use of session time. For the control group (Group B), SGDs led by assigned facilitators followed a general discussion and question-answer format based on predetermined topics.

We conducted pretests (initial 10 min after briefing about group dynamics, mind map creation) and posttests for both groups using a prevalidated questionnaire to gather feedback. These tests included 10 questions worth 10 marks, comprising single-response, multiple-response, problem-solving, and reason-assertion questions. The feedback questionnaires for both groups collected quantitative data on the quality of teaching and methods used in tutorials through a five-point Likert scale and qualitative data through open-ended questions.

Due to coronavirus disease 2019 restrictions, we collected feedback online from 11 randomly selected students for a planned focus group discussion covering themes on the relevance and execution of SGDs, prediscussion preparation by students, and the effects of division, discussion, and presentation in small groups.

Statistical analysis

We used descriptive statistics, mean and standard deviation, and paired t-test for pre- and posttest scores to assess significance, with p < 0.05 considered significant. The effectiveness of the intervention was measured using the class-average normalized gain (“g”), with a gain of more than 0.7 indicating high effectiveness, between 0.3 and 0.7 indicating medium effectiveness, and below 0.3 indicating low effectiveness. Feedback data were analyzed using percentages for quantitative data and coding and categorization for qualitative data, including online feedback.

## Results

In this study, we enrolled three batches of first-year MBBS students, totaling 125 participants, divided into 42, 42, and 41 students. These students were equally divided between the intervention (Group A) and control (Group B) groups for both the hematology and muscle nerve physiology themes, with 46 students in each group for hematology and 54 for muscle nerve physiology.

The learning outcomes between the intervention group (Group A using the 3D method) and the control group (Group B) were compared using pretests and posttests. There was a significant difference in performance between the groups in both the hematology and muscle nerve physiology themes. Specifically, the intervention group demonstrated a larger effect size of 1.55 in hematology and 1.4 in muscle nerve physiology, indicating a more substantial improvement in learning outcomes than the control group. These findings are detailed in Table 1 for hematology and Table 2 for muscle nerve physiology.

**Table 1:** Hematology pretest and posttest scores SD, Standard deviation

Group	Subgroup	Mean ± SD	95% confidence interval	p-value	Effect size (Cohen’s d)	Class-average normalized gain (“g”)
Control	Pretest	5.91 ± 2.12	(5.297, 6.522)	p < 0.05 (significant)	0.22 (small)	0.1
	Posttest	6.36 ± 1.91	(5. 808, 6.912)			
Intervention	Pretest	5.80 ± 1.84	(5.268, 6.332)	p < 0.05 (Significant)	1.55 (large)	0.6
	Posttest	8.41 ± 1.51	(7.973, 8.846)			

**Table 2:** Muscle nerve physiology pretest and posttest scores SD, Standard deviation

Group	Subgroup	Mean ± SD	95% confidence interval	Significance "p"	Effect size (Cohen’s d)	Class-average normalized gain "g"
Control	Pretest	5.96 ± 2.55	(5.279, 6.640)	p < 0.05 (significant)	0.77 (medium)	0.4
	Posttest	7.64 ± 1.69	(7.189, 8.090)			
Intervention	Pretest	5.85 ± 2.46	(5.193, 6.506)	p < 0.05 (significant)	1.4 (large)	0.6
	Posttest	8.61 ± 1.31	(8.260, 8.959)			

The class-average normalized gain “g” was low (0.1) for the control group but medium (0.6) for the intervention group in hematology, and similarly, “g” was medium (0.4) for the control group compared to 0.6 (medium) for the intervention group in muscle nerve physiology, suggesting that the intervention method was more effective in enhancing learning.

Feedback questionnaires revealed that 61% of the intervention group agreed there was adequate interaction in the hematology section (Table 3), and 87% strongly agreed in the muscle nerve physiology section compared to the control group (Table 4). Additionally, 37% of the intervention group agreed that the SGD enhanced communication skills in the hematology section, and 52% strongly agreed in the muscle nerve physiology section. The optimal group size was considered satisfactory by 65% of participants in the hematology section and 57% in the muscle nerve physiology section. Learning objectives set at the beginning of sessions were agreed upon by 41% of the intervention group in hematology and strongly agreed upon by 48% in muscle nerve physiology. Clarification of doubts and misconceptions was strongly agreed upon by 61% in the intervention group for hematology and 52% in muscle nerve physiology. Session summarization was agreed upon by 57% in the hematology section of the intervention group and strongly agreed upon by 81% in the muscle nerve section. Additional insights from the study regarding communication skills, interaction, and the effectiveness of small-group sizes are further elaborated in Tables 5-7, including open-ended responses and feedback from both groups.

**Table 3:** Hematology feedback on closed-ended questions

Group	Feedback statement	Strongly agree (%)	Agree (%)	Neutral (%)	Disagree (%)	Strongly disagree (%)
Control group	Adequate interaction	20 (44)	24 (52)	2 (4)	0	0
	Enhanced communication skills	24 (52)	16 (35)	6 (13)	0	0
	Complemented the knowledge learned in lectures	28 (61)	18 (39)	0	0	0
	Optimal group size	21 (46)	3 ( 6)	7 (15)	15 (33)	0
	Learning objectives set at the beginning	23 (50)	17 (37)	6 (13)	0	0
	Assured clarification of doubts and misconceptions	26 (57)	17 (37)	1 (2)	2 (4)	0
	Session was summarized	20 (44)	17 (37)	7 (15)	2 (4)	0
Intervention group	Adequate interaction	13 (28)	28 (61)	5 (11)	0	0
	Enhanced communication skills	23 (50)	17 (37)	6 (13)	0	0
	Complemented the knowledge learned in lectures	28 (61)	15 (33)	3 (6)	0	0
	Optimal group size	15 (33)	30 (65)	1( 2)	0	0
	Learning objectives set at the beginning	21 (46)	19 (41)	6 (13)	0	0
	Assured clarification of doubts and misconceptions	28 (61)	15 (33)	3 (6)	0	0
	Session was summarized	18 (39)	26 (57)	2 (4)	0	0

**Table 4:** Muscle nerve physiology feedback on closed-ended questions

Group	Feedback statement	Strongly agree (%)	Agree (%)	Neutral (%)	Disagree (%)	Strongly disagree (%)
Control group	Adequate interaction	15 (28)	29 (53)	8 (15)	2 (4)	0
	Enhanced communication skills	12 (22)	23 (43)	17 (31)	1 (2)	1 (2)
	Complemented the knowledge learned in lectures	17 (31)	30 (56)	6 (11)	1 (2)	0
	Optimal group size	14 (26)	29 (54)	0	11 (20)	0
	learning objectives set at the beginning	20 (37)	30 (56)	4	(7)	0
	Assured clarification of doubts and misconceptions	15 (28)	32 (59)	5 (9)	2 (4)	0
	Session was summarized	10 (19)	25 (46)	15 (28)	3 (5)	1 (2)
Intervention group	Adequate interaction	47 (87)	7	(13)	0	
	Enhanced communication skills	28 (52)	25 (46)	1 (2)	0	0
	Complemented the knowledge learned in lectures	45 (83)	9	(17)	0	0
	Optimal group size	31 (57)	23 (43)	0	0	0
	learning objectives set at the beginning	26 (48)	26 (48)	2 (4)	0	0
	Assured clarification of doubts and misconceptions	28 (52)	24 (44)	2 (4)	0	0
	Session was summarized	44 (81)	8 (15)	2 (4)	0	0

**Table 5:** Blood and muscle nerve physiology feedback on open-ended questions among the control group participants SGD, Small-group discussion

Control group	Coding and categorization	Excerpts from open-ended responses
Fallacies	Group size	“Size of the group is big”
		“Group size is big and many students are not showing interest”
	Student interaction	“There was no interaction among students and sharing of knowledge”
		“Could have been more interactive involving students”
Suggestions	Preparation of students	“Students should come prepared for SGD”
		“I think that coming to SGD having read the topics would be very useful to us”
	Division into small groups	“All students should be divided into small groups and should discuss a topic”
		“Group should be small. All students should take part”
	Discussion	“During SGD, only questions were asked on the topics rather than discussing the topics”
		“The time provided should be divided into two halves: one for discussing the topics in brief. The next half should be questioning the students and knowing which places they lag behind and providing them with sufficient assignments”
	Summarization	“Teachers should summarize at last”
	Positive teacher interaction	“Teachers cleared each and every doubt and briefed on topics nicely”
		“There was adequate interaction between students and teachers”
		“I appreciate the efforts of our teachers for the same”
	Pre and post tests	pre and post test should be continued”
		“Pre- and posttest evaluations were very much helpful in evaluating ourselves in grading the importance of SGD’s and in the understanding of many topics”
	Use of media	"Video presentation if included would be more interactive”
		“If blackboard is used, then student can also write important points in their copy”

**Table 6:** Blood and muscle nerve physiology feedback on open-ended questions among the intervention group participants SGD, Small-group discussion

Coding and categorization	Excerpts from open-ended responses
Distribute	“Division helped to cover the topics in the specified time”
	“Provides an opportunity to discuss the topics with each other”
	“Best part is the small groups. These small groups help to remove hesitation”
	“Small groups make it more interactive”
	“Enhanced knowledge about topics that were divided”
Discussion	“By teaching others and increasing their knowledge, the student can understand the topic much deeper and better”
	“Summarizes the topic in not just ours but also by others (i.e., by a different point of view)”
	“Got to know many more information of topics as we discussed among ourselves”
	“Group discussion helps in better doubt clearing, and time given in early part to discuss is better for conceptualization”
	“Allowed us to revise the topics and provided the platform to explain so as to allow retention of concepts”
	“While preparing with our groups, it enhanced the way we exchanged our knowledge”
Development	“Many doubts are cleared and knowledge is enhanced”
	“It enhanced communication skills. Summarization of previously taught topics proved essential”
	“Doubts were cleared, excellent SGD, enhanced our knowledge”
	“Getting better clarity on topic and environment was friendly to explain our views and summarization of topic, and there
	was improvement in our communication skills”
	“Improved our presentation skills”
	“Blackboard presentation, good pretest and posttest helped a lot”
	“It is very beneficial because a lot of the topics are summarized and explained in a very short amount of time”
	“The extra points added by teacher and extra questions digs in deeper to understand the topics so that students can
	recollect and familiarize with way one would understand questions”
	“SGD provided an opportunity to awaken the teacher inside a student”
	“Practice of chalk and board method frees from stage fear and boast’s confidence”
	“Interactive discussion makes learning fun and makes us remember concepts better, boosts confidence”
	“We studied a large amount of topic in a short period of time, which for me is a great thing”
	“Such SGDs should be frequently taken, it is ‘study with enjoyment’”
	“This SGD increased my clarity on the topic as it involves opinions of many students and teachers and clarifying the
	doubts asked by many students”
	“increased self-confidence”

**Table 7:** Excerpts from the online discussion based on themes SGD, Small-group discussion

Themes	Excerpts from participant responses
Theme 1: Relevance of SGD as a teaching-learning method in preclinical sciences	“Personally, I believe that SGD is indeed an inevitable way of teaching-learning. It helps in maximum exchange of facts between individual with minimum implication of time; involvement of faculties helps clarify misconceptions regarding a topic or clarifies doubts”
	“As it is said modern problems require modern solutions; therefore, the concept of SGD is revolutionizing student’s learning approach toward the preclinical sciences. The essence of these discussions lies in the belief that mentors are presenting the facts and the students are devising their ways of proposing the same theory in a slightly unique way, creating a space where every intellectual is getting an opportunity to hone his/her understanding level”
	“It lets to know about one’s mistakes and weaknesses”
	“Different students have different ways of understanding and analyzing a given topic which gets shared during discussion, helping to broaden up one’s thinking and perception toward the topic”
Theme 2a: Comment on the method in which it was conducted (traditional question and answer method)	“Here, all students were randomly asked questions. They had to answer quickly on the spot. It enables quick thinking and ‘recollecting all ideas’ for the question”
	“It does not give wide range of scope in group discussion. Also, in this method, it doesn’t provide an opportunity to represent the presentation skill of student”
	“Traditional method looks like just another lecture by the faculty members”
Theme 2b: Comment on the method in which it was conducted (interventional small-group method)	“SGD method is better in the viewpoint that since students are encouraged to individually present a topic, they are boosted up with better communication skills and confidence”
	“Small-group method is very useful. It saves time and concepts are understood in a very fun and easy way. In comparison with traditional method, this method is very interactive and knowledgeable. It also boosts the confidence of the speaker who explains the topic on board”
	“SGD is highly appreciable. It enables us to have a better understanding because I can’t teach someone if I don’t have a strong basics of the topic”
	“Group discussion was better as retaining power has been increased. It was more student centered and student friendly. The chalk and board presentation was more conclusive, and now whenever we study that topic again, it helps to remind/recollect that points quickly. We got the opportunity to discuss, debate, and solve problems and reflect the concepts”
Theme 3a: Prediscussion preparation on the part of students: reluctance effects versus benefits	“It helps us in going through that topic thoroughly. It also helps us to come across some doubts while preparing, which when asked to teachers, our concepts gets crystal cleared.”
Theme 3b: Effects of division and discussion in small-group method	Division of topics is a great time saver of course”
	“Discussion definitely promotes communication, teamwork; in context of topics, it helps in diversifying the knowledge existing within the students, understanding the concepts with clarity”
	“Due to division and discussion of topics in small groups, the topics are better understood by students as they are discussed in simple language and later explained by the teacher. This helped students in easy grasping and better retention”
	“Due to division of topics in SGD, each group could focus on the particular topic and clear all the doubts related to that topic by discussing among themselves”
	“Participants refers to various other sources before discussing a topic which adds up to the knowledge of other members taking part in the discussion”
	“Division of topics among students in collaboration speeds up/boosts the learning process”
Theme 3c: Effects on development in students because of division and discussion and presentation	“It helps in developing stage confidence in presenting the topic, also helps in getting familiar with the topic, so that the confidence on a particular topic/ chapter escalates. I strongly recommend this method of group discussion that is a small-group method for discussion”
	“The group discussion helped us in improving our listening and our communication skills. This whole thing helped us to learn how to work as a team which is of great importance to us in our future”
	“The knowledge and mental horizon of the students become broadened. Division enables the reduction in burden of course. It provides enough time for students and hastens the preparation process. Discussion enables to share the thoughts and concepts. Presentation further organizes the thought process of students, as students sequentially build up the concepts not in a haphazard manner”
Theme 4: Suggestions/recommendations to enhance small-group discussions	“After every groups’ explanation on the board, the person explaining should ask one question each to the other groups relating to the topic he/she explained”
	“Topic division should be much more in number so that every student should get a topic”
	“More time can be given for such discussions”
	“More topics can be discussed especially those topics which are difficult to understand by the students”
	“Clinical approach should also be done by investigations on clinical case studies”

## Discussion

Physiology is a complex discipline where concepts, terms, and structures are often challenging to visualize and understand [6,7]. Effective teaching and learning strategies are crucial for students to comprehend these concepts, especially as they relate to pathology when medical students begin interacting with patients [8]. With rapid increases in physiology knowledge, teachers and students face difficulties coping with the content’s complexity, particularly as student numbers increase [9,10]. The present study aimed to evaluate whether modifications in SGDs could improve learning outcomes compared to traditional group discussions.

Previous studies, such as those by Srivastava et al. [10], highlighted the statistical significance of pretest and posttest scores in traditional and interactive intragroup tutorials, supporting our findings where modified SGDs demonstrated improved learning outcomes. Similar findings were noted in a study by Arja et al. [11], where students' performances improved significantly in the posttest after the group discussion sessions. Our study further revealed that SGDs could enhance communication skills, a finding echoed by Walton [12], who noted that group methods make students more articulate and better public speakers.

The consensus among participants that small-group tutorials enhance information retention aligns with findings by de Jong et al. [13-14] and Pal et al. [15-16] who concluded that small-group teaching-learning methods promote self-directed learning and positively impact student learning. Our study also identified the optimal group size for SGDs, emphasizing the importance of small groups in removing hesitation and enhancing interactivity, a critical aspect of effective learning environments.

Our study had several important limitations. First, the study’s single-masked design could introduce biases if the facilitators inadvertently influenced student performance or feedback. Second, the study relied on voluntary participation from first-year MBBS students, potentially introducing selection bias toward more motivated individuals who might be more receptive to the 3D method. Third, the intervention and control groups were derived from the same cohort, raising the possibility of cross-group communication that could affect the study’s outcomes. Additionally, the study was conducted within a single academic institution, limiting the generalizability of the findings to other settings with different educational cultures or resources. Finally, the use of pre- and posttests as the primary outcome measures might not fully capture the long-term retention of knowledge or the development of critical thinking and problem-solving skills that are essential components of medical education. Future studies could address these limitations by incorporating a double-masked design, expanding the participant pool across multiple institutions, and employing longitudinal follow-up to assess the sustained impact of the 3D method on medical education.

## Conclusions

Our study demonstrates that the 3D method significantly improves learning outcomes in medical education. This approach enhances student interaction, communication skills, and comprehension of complex physiological concepts, thereby promising to enrich the educational experience in medical schools. Implementing the 3D method makes SGDs more student-centered and interactive by organizing larger groups into smaller subgroups and engaging in discussions and presentations. The results indicate that students’ performance markedly improves following the 3D intervention. Furthermore, the 3D method boosts students’ communication skills and fosters a stronger relationship between the facilitator and the students.
